# Measurements of face mask’s capability to block ionizing radiation

**DOI:** 10.1038/s41598-025-89643-4

**Published:** 2025-02-22

**Authors:** Hsingtzu Wu, Hong-Da Liu, Tzu-Hsiang Lin, Ming-Wei Lin

**Affiliations:** Institute of Nuclear Engineering and Science, National Tsing Hua University, 300044 Hsinchu, Taiwan ROC

**Keywords:** Health care, Energy science and technology, Materials science

## Abstract

Experts suggest wearing a face mask during a radiation emergency if it is impossible to get inside immediately and high-level protective respirators are unavailable. This study quantitatively investigated seven face mask materials’ ability to block radioactive alpha and beta radiation. Rayon fiber, pure cotton, paper fiber, polyester fiber, nonwoven fiber, advanced nonwoven fiber, and N95 were examined. The results suggest that the abovementioned mask materials can block more than 90% of alpha particles. Rayon fiber, polyester fiber, and N95 can block almost all radioactive alpha particles. On the other hand, the measurements suggest that all tested materials could not effectively block beta particles. Polyester fiber and N95 block more than 10% of beta particles, which outperform other mask materials. In addition, the results imply that the electret fiber might help block beta particles. This study suggests that wearing a relatively thick polyester or N95 mask may be a better choice than wearing a thin nonwoven mask to prevent inhaling alpha and beta particles during a radiation emergency.

## Introduction

Nations worldwide have committed to reducing greenhouse gas emissions to curb their impact on climate. Nuclear power has drawn attention as one key contributor to the low-carbon-emission energy system, and 31 countries have signed declarations to triple nuclear generation by 2050^1–3^. The likelihood and potential impact of nuclear accidents has been a critical factor in public concern about nuclear facilities^4^. A radiation emergency is almost always accompanied by a nuclear accident, and it may increase individuals’ exposure to ionizing radiation^5^. Ionizing radiation is categorized into alpha particles, beta particles, and gamma rays. The first two were considered in this study because gamma rays require substantial, dense shielding for effective blockage. Alpha particles are brought to rest by a few centimeters of air or less than a tenth of a millimeter of biological tissues, and they can cause multiple ionizations within a very small distance. This allows them to cause more biological damage for the same amount of deposited energy^6^. On the other hand, beta particles can travel through many centimeters or even meters in the air and through millimeters of skin or tissues. Sufficient intensity of beta radiation can damage internal cells and organs^7^. Therefore, it is desirable to prevent alpha and beta particles from entering human bodies. Face masks protect the wearers from contact with droplets and sprays that may contain germs and lower the risk of respiratory virus transmission during a pandemic. They may be used to shield radioactive materials that emit alpha or beta particles. The National Council on Radiation Protection and Measurements suggests that workers and volunteers responding to a nuclear detonation incident outside the affected area may use face masks as minimal protection and source control devices against respiratory hazards when high-level protective respirators are unavailable^8^. The National Safety Commission (Taiwan) gives more detailed instructions on when to use a full facepiece respirator, N95, or ordinary face mask^9^. U.S. Centers for Disease Control and Prevention suggests that the public cover their mouths and noses with a mask to reduce the amount of radioactive material they breathe during a radiation emergency when they can’t get inside immediately^10^. Alexander suggested that most air-purified masks afford excellent protection from inhalation of radioactive material^11^. Comprehensive investigations of the properties of face masks have been conducted. The National Institute for Occupational Safety and Health (NIOSH) tests and certifies respirators based on their filtration capabilities and efficiency levels under various environments^12^. Research on face masks has focused on measuring their filtration efficiencies in preventing airborne transmission^13,14^. However, quantitative information about the capability of mask materials to block ionizing radiation is unavailable. This study aimed to fill the gaps by testing the shielding properties of seven mask materials (Rayon fiber, pure cotton, paper fiber, polyester fiber, nonwoven fiber, advanced nonwoven fiber, and N95).

## Results and discussion

**Fig. 1:**
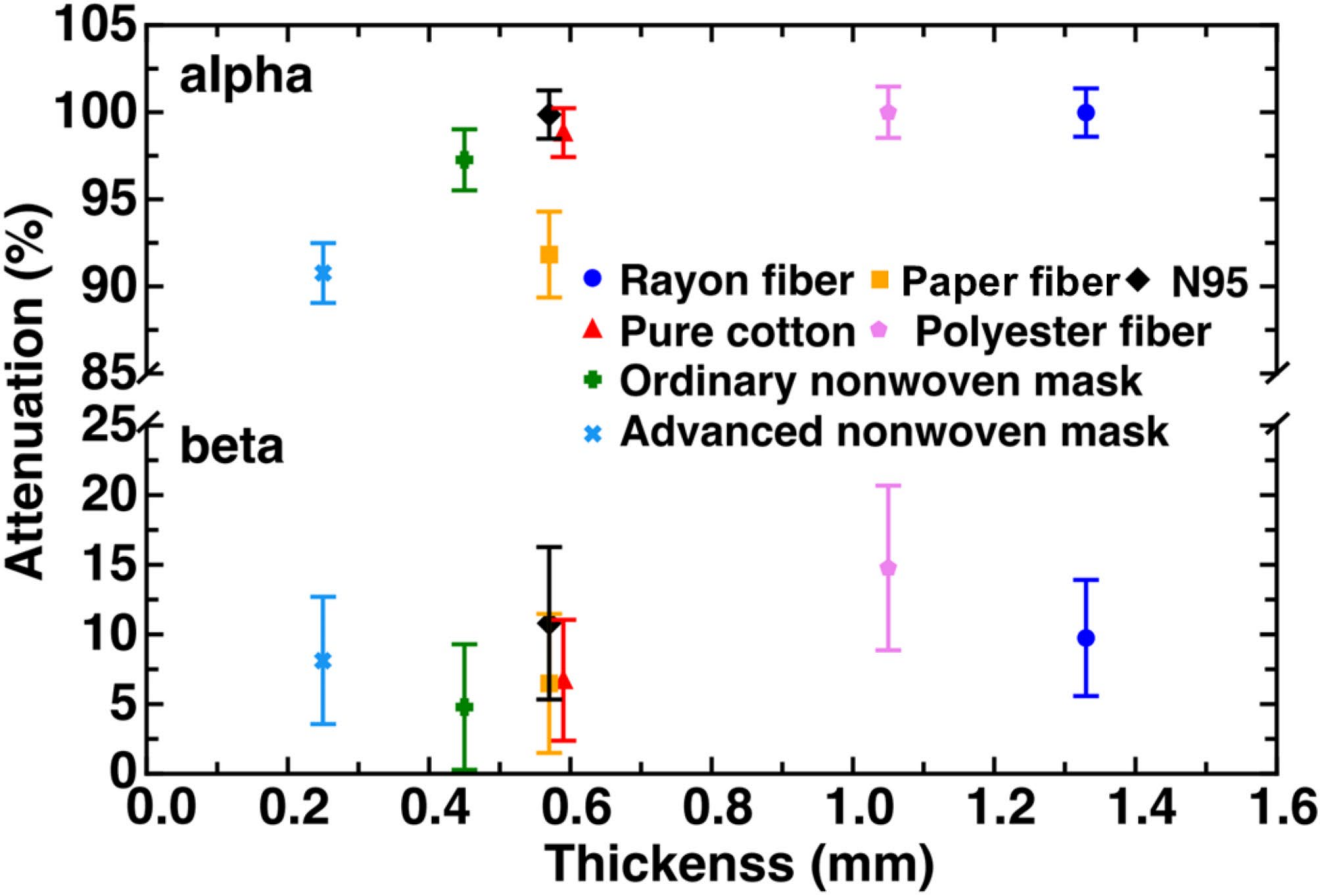
Mask materials can block alpha particles (upper section) and beta particles (lower section). A larger *Attenuation* percentage implies better shielding capability.

Seven mask materials’ capability to block alpha and beta particles is estimated by *Attenuation*, the difference in the amount of radiation detected with and without shielding of the mask materials. *Attenuation* was computed using

1 $$\:\text{A}\text{t}\text{t}\text{e}\text{n}\text{u}\text{a}\text{t}\text{i}\text{o}\text{n}=\frac{{\stackrel{-}{{x}_{e}}}_{no\:shielding}-{\stackrel{-}{{x}_{e}}}_{shielded}}{{\stackrel{-}{{x}_{e}}}_{no\:shielding}}\times\:100\%,$$

where

2 $$\:\stackrel{-}{{x}_{e}}=\frac{\sum\:_{i=1}^{N}{x}_{i}}{N}$$

,where $\:{x}_{i}$ is the counting rate per 20 s, and $\:N$ is 20 in this study. The measurements were conducted several times, and Fig. 1 shows the results. A larger *Attenuation* percentage implies better shielding capability. Deviations of all measurements are not significant at *p* < 0.01 (p was estimated with^15^). The error bars in Fig. 1 show the standard deviations of *Attenuation* for different materials ($\:{{\upsigma\:}}_{\text{m}\text{a}\text{t}\text{e}\text{r}\text{i}\text{a}\text{l}}$​) values that considered error propagation. Standard deviations were calculated using

3 $$\:{{\upsigma\:}}_{\text{m}\text{a}\text{t}\text{e}\text{r}\text{i}\text{a}\text{l}}=\frac{\sqrt{{{\upsigma\:}}_{\text{s}\text{h}\text{i}\text{e}\text{l}\text{d}\text{e}\text{d}}^{2}+{{\upsigma\:}}_{\text{n}\text{o}\:\text{s}\text{h}\text{i}\text{e}\text{l}\text{d}\text{i}\text{n}\text{g}}^{2}}}{{\stackrel{-}{{x}_{e}}}_{no\:shielding}}\times\:100\%,$$

where

4 $$\:{\upsigma\:}=\sqrt{\sum\:_{i=1}^{N}\frac{{x}_{i}-\stackrel{-}{{x}_{e}}}{N}}\times\:100\%$$.

**Fig. 2:**
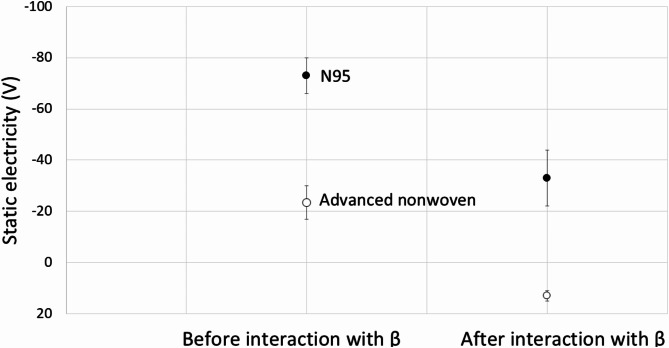
The static electricity of N95 and advanced nonwoven masks is reduced after interaction with beta particles, interpreted as a result of interactions with the beta particles. It suggests that the electret fibers may help shield beta particles.

The results suggest that all materials can block more than 90% alpha particles. The N95 mask, Rayon fiber, and polyester mask could effectively block almost all alpha particles. Even though regular paper is reported to be sufficient to stop alpha particles^16^, our measurements show that paper fiber blocks about 90% of alpha particles. It is explained that the pore size of the latter is larger to enhance breathability while sacrificing shielding capability. On the other hand, none of the tested materials could effectively block beta particles. Polyester fiber and N95 block more than 10% of beta particles, which outperform other mask materials. Rayon fiber, polyester fiber, and N95 block more beta particles than nonwoven masks. In addition, Fig. 1 shows that the advanced nonwoven mask blocks more beta particles than the ordinary nonwoven mask. Both were made of three-layered nonwoven fabric, while the former was thinner with an electret fiber. In general, a thicker material shields better. Therefore, it suggested that the difference may be related to the electret fiber because beta particles are essentially electrons. Figure 2 shows the static electricity of two materials with electret fibers (N95 and advanced nonwoven masks) before and after shielding beta particles. The static electricity was measured using the high precision mode (± 10 V) of the KEYENCE static electricity sensor (SK-H050) at 27.2 ± 1℃ and with 68 ± 5% relative humidity. The static electricity sensor, with a response time of 0.8 s, automatically measured data every 1.4 ms for each sample. Each sample was measured for about 3 s. Figure 2 shows the maximum, minimum, and average values of the measurements. The solid and open circles in this figure indicate data for N95 and the advanced nonwoven masks, respectively. The static electricity in both cases is reduced after interactions with beta particles, interpreted as a result of collisions with the beta particles. In other words, it suggests that the electret fibers may help shield beta particles. On the other hand, Fig. 1 shows that the advanced nonwoven mask shielded alpha particles less efficiently than the ordinary one. It suggests that an electret fiber may not help shield alpha particles.

During a radiation emergency, the radiation dose should be kept to a level that is as low as reasonably achievable with principles of less time spent near radioactive sources, greater distance from the sources, and behind shielding from the sources^17^. Experts suggest wearing a face mask during a radiation emergency if it is impossible to get inside immediately and high-level protective respirators are unavailable. This study compares seven face materials’ capability to block alpha and beta particles. The results suggest that wearing a relatively thick N95 mask or a mask made of rayon fiber or polyester fiber may block alpha and beta particles more efficiently than wearing a thin nonwoven mask in cases where alpha or beta emitters may be brought near a person during a radiation emergency. In addition, a layer of electret fiber in the face mask may help shield beta particles. Note that prolonged usage of a face mask during a radiation emergency should be avoided. It is reported that using a disposal face mask for several hours may create a microclimate that plays a crucial role in the growth of fungal and bacterial colonies^18^. In addition, radioactive materials may accumulate on the mask’s texture, and the effect of an electret fiber would be weakened after some time as the static electricity decreases after interactions with beta particles. While face masks made of rayon or polyester fiber are reusable under normal conditions, we suggest they should be discarded after use in a radiation emergency.

## Materials and methods

**Fig. 3:**
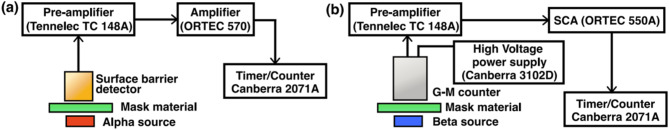
Schematic diagrams of the experimental setup for (a) alpha particle detection and (b) beta particle detection.

**Fig. 4:**
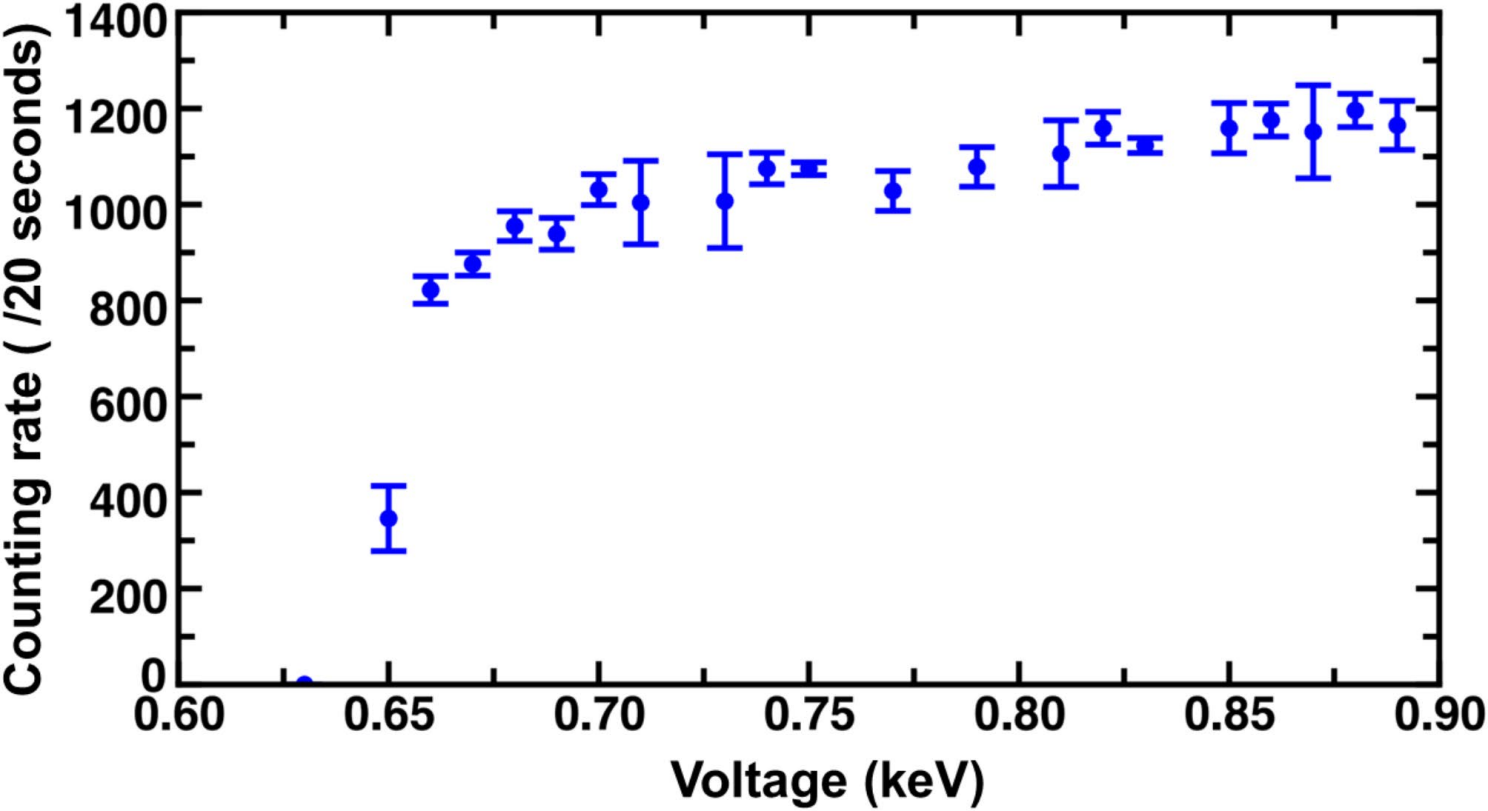
Counting plateau for the G-M counter.

The alpha source used in this study was a mix of Am-241, Cm-244, and Pu-239; the beta source was Sr-90. A surface barrier detector and a Geiger-Mueller (G-M) counter were used for alpha and beta detection, respectively. The surface barrier detector and G-M counter are widely used for measuring the activities of alpha and beta sources due to their high sensitivity and efficiency. The surface barrier detector is a silicon-based semiconductor detector with excellent efficiency of charge collection and accuracy of measurement. The detection efficiency of the G-M counter for charged particles is nearly 100%, as any ion pair formed within the fill gas can trigger a full discharge reaction. More information on the radiation detectors can be found in^19^. In addition, Oxford Tennelec TC 148 A Preamplifier, ORTEC Model 570 Amplifier, ORTEC Model 550 A Single-Channel Analyzer (SCA), Canberra Model 3102D High Voltage Power Supply (accuracy of 0.01 kV), and two Canberra Model 2071 A Dual Counter/Timers were used in the experiments. Figure 3 sketches the experimental setup. Signals below 0.2 V were filtered via the SCA, and the number of counts per 20 s was recorded. The G-M counter should be operated around the center of the counting plateau to ensure the reliability of the measurement. The counting plateau was determined using counting rates at various voltages, as shown in Fig. 4, where the experimental mean values are computed using Eq. (2) and the error bars are standard deviations calculated using Eq. (4). The experimental data using the G-M counter shown in the previous section were collected at 0.79 kV.

**Fig. 5:**
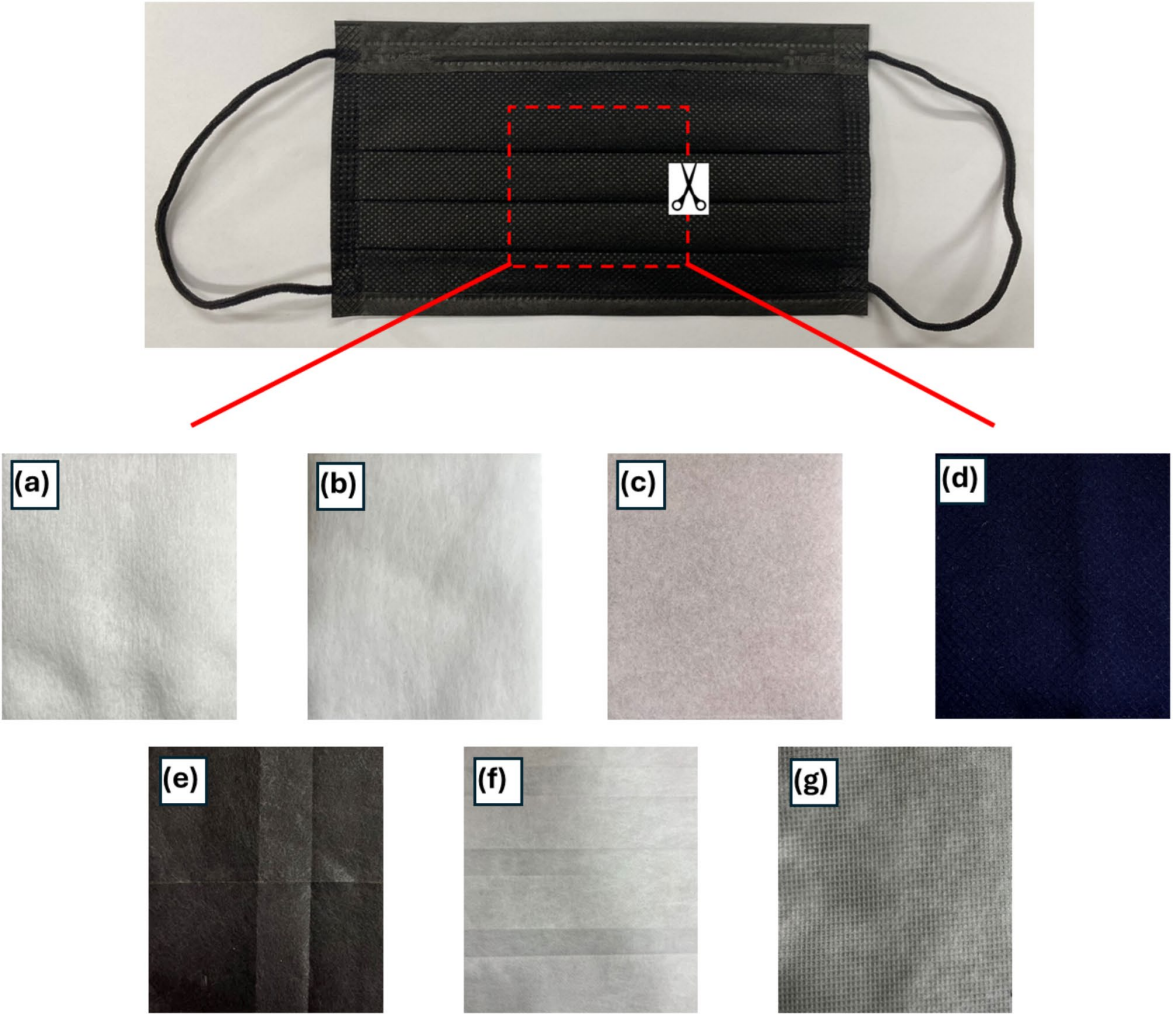
Captured images of various mask materials. (a) Rayon fiber, (b) Pure cotton, (c) Paper fiber, (d) Polyester fiber, (e) Ordinary nonwoven, (f) Advanced nonwoven, and (g) N95. Note that the rayon fiber, pure cotton, and paper fiber were cosmetic pads that could be used to make face masks. Polyester fiber, nonwovens, and N95 were cut from face masks.

All experiments were performed at room temperature and ambient pressure in the air medium. Counts per 20 s were recorded ten times for eight conditions. In one condition, the bare radioactive sources were used. In other condition, radioactive sources were covered with seven different types of mask materials. All mask materials were purchased in local chain stores, and it is assumed that they were mass productions with the standard technique. Three shields were cosmetic cotton pads made of Rayon fiber (0.1432 g/cm^3^), pure cotton (0.1697 g/cm^3^), and paper fiber (0.0660 g/cm^3^). These samples were raw materials with simple machining, such as cutting and edge-pressing. The other four were pieces cut from face masks from local chain stores. The first one was named the “ordinary nonwoven mask” with a density of 0.1938 g/cm^3^. It was made of three-layered nonwoven fabric. The outer layer was nonwoven polypropylene fabric, the middle was melt-blown with BFE > 99%, and the one that would touch a mask wearer’s face was polyethylene and polypropylene nonwoven fabric. It had a certification of CNS 14,774. The second face mask was made of three-layered nonwoven fabrics as well. The outer and inner layers were similar to the ordinary nonwoven mask. The middle layer was HEPA nonwoven fabric (BFE/PFE/VFE > 99%). It has certifications in CNS 14,774, QMS 2148, MD 6,131,000,263, and ISO 13485:2016. The weight was 20% less than the original nonwoven mask. Therefore, it is called the “advanced nonwoven mask,” and its density was 0.2313 g/cm^3^. The third type was an N95 mask with a density of 0.2474 g/cm^3^. In addition to the three nonwoven fabrics, it included a layer of composite fibers. It had certifications of CNS 14,774, CNS 14,755, ASTM F2100:2020, 42 CFR Part 84, QMS 5329, MD 6,107,000,546, and Nelson Lab 1,530,607-S01. The advanced nonwoven mask and N95 mask use electret fiber to attract small particles with electric interaction. The fourth was a mask made of 100% polyester fiber; its density was 0.2592 g/cm^3^. Figure 5 shows the pictures of the abovementioned mask materials. The size of the detectors determined the size of the samples, which was sufficient to cover the radioactive sources. Note that the rayon fiber, pure cotton, and paper fiber were cosmetic pads that could be used to make face masks. Polyester fiber, nonwoven fibers, and N95 were cut from face masks.

## Data Availability

Raw data are available from the corresponding author upon reasonable request.
